# CRISRP/Cas9-Mediated Targeted Mutagenesis of Tomato Polygalacturonase Gene (*SlPG*) Delays Fruit Softening

**DOI:** 10.3389/fpls.2022.729128

**Published:** 2022-05-19

**Authors:** Hongmei Nie, Yu Shi, Xueqing Geng, Guoming Xing

**Affiliations:** ^1^College of Horticulture/Collaborative Innovation Center of Improving Quality and Increasing Profits for Protected Vegetables in Shanxi, Shanxi Agricultural University, Taigu, China; ^2^School of Agriculture and Biology, Shanghai Jiao Tong University, Shanghai, China

**Keywords:** tomato, polygalacturonase, CRISPR/Cas9, genome editing, fruit softening

## Abstract

Polygalacturonase (PG) gene has been documented as a key candidate for the improvement of fruit firmness, which is a target trait for tomato production because it facilitates transportation and storage. To reduce the expression of the *PG* gene, most of the elite commercial tomato varieties were obtained by RNA interference technology. However, this approach of producing commercialized tomatoes by integration of the exogenous gene is controversial. In this work, CRISPR/Cas9 technology was used to induce the targeted mutagenesis of the *SlPG* gene to delay the softening of tomato fruit. Results showed that the *SlPG* gene was frameshift mutated by 4 bp deletion, 10 bp deletion, and 1 bp insertion, which generated premature translation termination codons. Compared with wild-type (WT), homozygous T_1_-generation tomato plants exhibited late fruit softening under natural conditions. Consistent with this phenomenon, the firmness value of WT fruit was lower in *slpg* mutant fruit, and the physiological loss of water was higher. Collectively, these data demonstrate that the mutation of the *SlPG* gene delays tomato fruit softening. More importantly, 8 out of 20 transgene-free tomato plants, which were homozygous for null alleles of *SlPG*, were separated in the T_3_-generation of line *slpg*T_2_-#2. This transgene-free *slpg* may provide materials for more in-depth research of *SlPG* functions and the molecular mechanism of fruit softening in tomatoes.

## Introduction

Tomato (*Solanum lycopersicum*), one of the most significant commercial vegetable crops grown in the world, has abundant nutritive compounds and elements (e.g., lycopene, zeaxanthin, vitamin C, and potassium) for human health (Vincent et al., 2013). Fruit softening is the most common defect consideration of commercial importance and has tremendous associated costs in terms of fruit shelf life, frequency of harvest, transportation, and storage (Garcia-Gago et al., 2009). During fruit ripening, the biochemical and physiological processes are sophisticated, and the solubilization of the cell wall is caused by a group of enzymes (Payasi et al., 2009). One of the key enzymes is PG (EC 3.2.1.15), which functions in the breakdown of pectin, a polymer of galacturonic acid that forms part of the structural support of cell wall (Bird et al., 1988). In 1965, the PG enzyme was first reported in relation to the firmness of the tomato fruit (Hobson, 1965), and the *PG* gene sequence was first cloned from tomatoes in 1986 (Grierson et al., 1986). In 1988, with the application of molecular genetics, PG gene expression was first manipulated with transgenic approaches in tomatoes, where the suppression of *PG* gene expression resulted in significantly firmer fruit (Bird et al., 1988; Sheehy et al., 1988; Smith et al., 1988). Since then, the suppression of *PG* gene expression in other fruits, including strawberries (Quesada et al., 2009; Pose et al., 2013), pear (Zhang S. et al., 2019), and kiwifruit (Huang et al., 2020), have been reported and the desired traits were also obtained. Mostly, these fruits are reformed using antisense RNA (Smith et al., 1990; Ju et al., 1994; Kramer and Redenbaugh, 1994) or site-selected insertion (Cooley and Yoder, 1998) through transgene in elite commercial background varieties. Similar to other transgenic plants, the random integration of exogenous transgenes into the tomato genome may cause unstable and/or off-target effects, thereby causing public concern for human consumption and the commercialization of these tomatoes is restricted by complicated safety evaluation. Therefore, delayed tomato fruit softening that does not compromise exogenous genes is a key characteristic in modern tomato breeding.

In 2014, a developed technology, clustered regularly interspaced short palindromic repeat (CRISPR)/Cas (CRISPR-associated) (Doudna and Charpentier, 2014), as a robust and effective tool for the targeted gene of interest, has rapidly emerged in plant breeding with simple design and flexibility of operation (Xie et al., 2014; Zhang Y. et al., 2019). Since then, this system has been applied to generate and estimate targeted mutations in many representative plants, such as Arabidopsis (Li et al., 2013), tobacco (Nekrasov et al., 2013), rice (Shan et al., 2013), soybean (Michno et al., 2015), wheat (Upadhyay et al., 2013), and maize (Svitashev et al., 2015). CRISPR/Cas9-mediated genome editing in tomatoes was first successfully achieved in 2014 (Brooks et al., 2014) by designing two single guide RNAs (sgRNAs) to create mutagenesis in the *SlAGO7* gene. In 2017, the high rates of homozygous and biallelic mutants of *SlPIF4* were generated in the first generation by applying CRISPR/Cas9 technology, and the gene modifications were stably transmitted to the next generation (Pan et al., 2017). In 2018, the promoter of sgRNA, which provides a strong technical basis for creating mutagenesis in tomato, was optimized for this technology (Yan et al., 2018). In 2019, cytidine base editors, CRISPR/Cas9 derived tools, were applied in tomato successfully (Veillet et al., 2019), and the acetolactate synthase (ALS) gene was edited with the rate of 12.9%. These works have shown that CRISPR/Cas9 is a simple, efficient, and powerful gene editing tool in tomato molecular breeding.

In this study, CRISPR/Cas9 technology was used to induce the targeted mutagenesis of the *SlPG* gene in tomatoes. The use of the transient assay to select the high editing rate of sgRNA has not been reported previously. Out of 10 stable transgenic tomato events, 7 *slpg* mutants were obtained *via Agrobacterium*-mediated transformation. In T_1_-generation tomato plants, three homozygous for null alleles of *SlPG* frameshift mutated by short deletions (4 and 10 bp) and insertion (1 bp) exhibited late fruit softening under greenhouse conditions. The homozygous T_2_ *slpg* mutants, which have mutation types that were consistent with T_1_ generation, also exhibited late fruit softening. More importantly, in T_3_-generation tomato plants, “transgene-free” *slpg* mutants without any transgenic element were separated successfully. Such an approach is expected to reduce the deleterious effects caused by the random integration of the transgene into the tomato genome. These *slpg* mutants will provide materials for more in-depth research on improving tomato quality.

## Materials and Methods

### Plant Materials and Growth Conditions

The tomato cultivar Micro-Tom was used for transformation in this study. Micro-Tom refers to a miniature dwarf tomato that can grow at a high density, has a short life cycle, and can be transformed efficiently. It became a new model plant for functional genomic study. The origin, background, and biological characteristics of Micro-Tom were clear (Liu et al., 2008). WT Micro-tom (as a control) and all seeds collected from T_0_ mutant plants were sown under greenhouse conditions (16 h light/8 h dark and 22°C/18°C of day/night temperature, 80% humidity, and 10,000 lux light intensity).

### SgRNA Design and the CRISPR/Cas9 Vector Construction

**FIGURE 1:**
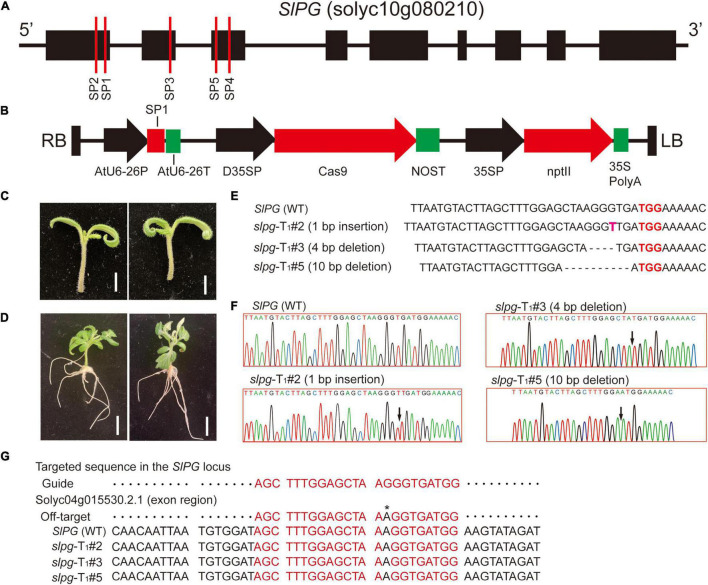
CRISPR/Cas9 technology was used to induce the targeted mutagenesis of the *SlPG* gene in tomatoes. **(A)** Schematic representation of tomato *SlPG* gene and target sites used in this study. Black boxes and lines represent exons and non-coding regions, respectively. Red vertical lines indicate the target site position and name of designed sgRNAs. **(B)** Schematic illustrating the pg1 vector. RB/LB, right/left border of T-DNA; AtU6-26P, *Arabidopsis* U6-26 promoter; SP1, sgRNA-SP1; AtU6-26T, *Arabidopsis* U6-26 terminator; D35SP, double cauliflower mosaic virus (CaMV) 35S promoter; Cas9, coding region of Cas9; NOST, Nos terminator; 35SP, 35S promoter; nptII, kanamycin resistance gene; 35S PolyA, 35S PolyA terminator. **(C)** Tomato seedlings were selected as explants used for transient transformation. Scale bar, 1 cm. **(D)** Well-developed hairy roots. Scale bar, 1 cm. **(E)** Sequences of WT plant and T1 homozygous *slpg* mutants (*slpg*-T_1_#2, *slpg*-T_1_#3, and *slpg*-T_1_#5) and representative mutation types induced at target site SP1 are presented, respectively. Nucleotides in red represent PAM sequences. Nucleotide in pink represents insertions and dashes between nucleotides represent deletions. **(F)** Sequence peaks of WT plant and T1 homozygous *slpg* mutants (*slpg*-T_1_#2, *slpg*-T_1_#3, and *slpg*-T_1_#5). The black arrowheads indicate the location of mutations. **(G)** Representative putative off-target site recognized by the gRNA designed in this study. One representative site was predicted by the website tool CRISOR. The sequence at the top of the alignment is predicted off-targeted sequence. Red sequences, nucleotides corresponding to the site recognized by the gRNA designed in this study; Black nucleotide with * on top, nucleotide mismatch in the sgRNA region.

The tomato gene *SlPG* sequence (GenBank Accession No.: NM_001247092) was first cloned and published in 1986 (Grierson et al., 1986) and its sequence information (solyc10g080210) was downloaded from *Solanum lycopersicum* ITAG3.2 of the Phytozome website^1^ (Goodstein et al., 2011). For the CRISPR/Cas9 vector construction, the sgRNAs used for genome editing were designed by using the website tool CRISPOR^2^ (Concordet and Haeussler, 2018). In this study, five sgRNAs that target the *SlPG* gene were selected and named SP1, SP2, SP3, SP4, and SP5. All the sgRNA sites were located at the front exons of the *SlPG* gene (Figure 1A). The sgRNA sequences were synthesized by Genewiz (Suzhou). For each sgRNA, a pair of DNA oligonucleotides was annealed to generate dimers, which were subsequently integrated upstream of the sgRNA scaffolds in the plasmid vector while expressing Cas9 and sgRNA. The sequence of Cas9 was codon-optimized for maize and assembled downstream of the double cauliflower mosaic virus (CaMV) 35S promoter together with a customized sgRNA driven by the AtU6-26 promoter. The kanamycin resistance gene, neomycin phosphotransferase II (*nptII*), driven by a CaMV 35S promoter, was selected as a plant screening marker. The schematic of the *SlPG*-CRISPR/Cas9 vector is shown in Figure 1B. In this study, *SlPG*-CRISPR/Cas9 vector containing sgRNA SP1, SP2, SP3, SP4, and SP5 was named pg1, pg2, pg3, pg4, and pg5, respectively. The schematic of pg1 vector is shown in Figure 1B.

### Transient Transformation of CRISPR/Cas9 in Tomato and Validating sgRNA Editing Activity

To test sgRNA editing activity, the constructed vectors (pg1, pg2, pg3, pg4, and pg5) were mobilized into *A. rhizogenes* K599 *via* electroporation. Hairy root transformation was performed following the reference (Pavli and Skaracis, 2010) with minor revision. The healthy and well-developed seedlings were selected as explants for root transformation. The hypocotyl was cut with the sterile blade at the bottom of the seedling (Figure 1C) and immersed the whole seedling into the K599 suspension for 10 min. Inoculated seedlings (5–7 seedlings per petri dish) were placed on square dishes that contained a cocultivation medium used in stable transformation and placed vertically in the greenhouse for 2 days. The seedlings were transferred into growth boxes that contained seedling germination medium, supplemented with appropriate concentrations of cefotaxime and antibiotics to eliminate bacteria and select transformed roots. After 15 days, seedlings with well-developed hairy roots emerged (Figure 1D) and could be directly used for the next analysis. To evaluate the ratio of mutagenesis of *SlPG*, genomic DNA was extracted from the hairy roots of each plant, and then the regions spanning the target sites were amplified by PCR. The PCR products were purified and sequenced. Target mutagenesis can be viewed *via* sequence peaks overlapping at the target site.

### Stable Transformation of CRISPR/Cas9 in Tomato and Screening for Mutations by Sequencing Analysis

CRISPR/Cas9 expression vectors were individually transformed into *A. tumefaciens* EHA105 *via* electroporation. The tomato cotyledon was used for tissue culture and transformation explant according to the protocol previously reported (Mallikarjunaiah and Moudgalya, 2017). Genomic DNA was extracted from the leaves of each plant in the T_0_ generation, and transgenic plants were identified by PCR primer Cas9-F/R and then used for subsequent gene editing analysis using primer SP1-F/R (Supplementary Table 1). Different types of gene editing can be identified *via* sequence peaks. Subsequently, the homozygous mutant types were identified by sequence alignment with the WT plant sequence. This method was also used in the T_1_ and T_2_ generations.

### Potential Off-Target Analysis

Genomic DNA of three T_1_ homozygous *slpg* mutants and WT tomato plants was used to evaluate the off-target effect of sgRNA SP1 used in this study. Off-target sites were estimated by the web tool CRISOR (see footnote 2) (Concordet and Haeussler, 2018). PCR analysis was performed using primers specific to the targeted gene (Supplementary Table 1). The amplified products were sequenced and evaluated the off-target effect of the sgRNA SP1.

### RNA Isolation and qPCR Analysis of Gene Expression

The WT plants and T_2_ homozygous *slpg* mutants grown in a greenhouse were used to compare the expression levels of *SlPG*. Total RNAs were extracted from frozen tissue using *TransZol* by following the protocol provided by the product supplier (TransGen Biotech, Beijing, China). For reverse transcription, 2 μg of total RNA was used to synthesize first-strand cDNA using *TransScript*® II One-Step gDNA Removal and cDNA Synthesis SuperMix (TransGen Biotech, Beijing, China). For qRT-PCR, the reactions were performed using *TransStart*® Green qPCR SuperMix (TransGen Biotech, Beijing, China). The PCR amplification conditions begin with a denaturing step for 20 s at 95°C, followed by 40 cycles of 95°C for 5 s and a primer extension reaction at 60°C for 30 s. PCR products were monitored using Bio-Rad CFX connect (Bio-Rad). All PCR reactions were run with three biological replicates each. Data were analyzed using the 2^–ΔΔCt^ method. *SlActin* (solyc03g078400) was selected as the internal reference.

### Fruit Firmness Measures and Softening Analysis

Fruit firmness was measured using a GY-4 digital fruit hardness tester fruit sclerometer (ALIYIQI, China) according to a previously reported method (Yang et al., 2017). Fresh intact fruits from different developmental stages, including breaker stage (BR), pink stage (PK), light red stage (LR), and red ripe stage (RR), were collected. A total of 20 fruits from different plants were taken at each stage, and each stage fruit was tested twice at equidistant points along the equatorial plane of the fruit. The fresh weight and size of each fruit were also measured to calculate the physiological loss of water. For the fruit softening analysis, more than 20 fruits at the RR stage were collected, the surface was sterilized first, and then stored at room temperature (23–25°C and 55–60% relative humidity). Every 10 days, fruit firmness was measured, and visual softening and collapse of fruit were assessed (Nambeesan et al., 2010). A total of 20 fruits were taken from different plants at each stage for analysis.

### Statistical Analysis

All experiments were repeated three times independently, and all results are reproducible. Statistical analyses were performed using SPSS 19.0 software. Two-tailed Student’s *t*-test was applied to compare the significance of differences between different groups. The *P*-values less than 0.05 were recognized as significant. OriginPro8 was used for drawing box plots and histograms. Values represent mean ± standard error (SE; *n* = 20).

### Primer Sequences Used in This Study

The primer sequences used for screening the transgenic positive transformants in transient and stable transformation, detecting the mutation induced by CRISPR/Cas9, potential off-target analysis, identifying transgene-free *slpg* mutant lines, and qRT-PCR analysis are listed in Supplementary Table 1.

## Results

### Evaluation of the Genome Editing Efficiency of SgRNAs by Transient Transformation

Hairy roots that contain different sgRNAs (SP1, SP2, SP3, SP4, and SP5) grown to a length of 5–6 cm (Figure 1D) were harvested individually and mutations at the targeted sites were examined. According to the results (Table 1), 15 out of 20 hairy roots show gene editing by the sgRNA SP1, which was predicted to target the first exon of the *SlPG* gene. In terms of editing efficiency, the ratio of SP1 gene editing is relatively high and reached 75%. Subsequently, the vector pg1 was chosen for further stable transformation.

**TABLE 1:** Summary of mutations generated for each sgRNA.

Name for sgRNA	gRNA-PAM^a^	No. hairy roots sampled	No. hairy roots mutation detected	% mutations
SP1	5′-AGCTTTGGAGCTAAG GGTGA**TGG**-3′	20	15	75
SP2	5′-TGTACTTAGCTTTGGA GCTA**AGG**-3′	19	3	15.8
SP3	5′-ACTGAAATAGAAGATC TGCA**TGG**-3′	18	4	22.2
SP4	5′-GGAGGAACTATCAATG GCAA**TGG**-3′	16	6	37.5
SP5	5’-TTTCAGACTACAAAGA TAGA**AGG**-3′	18	0	0

*^a^The PAM sequences are highlighted in bold.*

### Targeted Mutagenesis of *SlPG* Induced by CRISPR/Cas9

The mutants of *SlPG* at the target sites were named *slpg*. The T_0_ transgenic line was identified, and 7 out of 10 had heterozygous-targeted mutations in *SlPG*. The site-directed mutagenesis of *SlPG* was also observed at the target site in the T_1_ generation. A total of five T_1_ plants that were homozygous of *SlPG* induced by CRISPR/Cas9 were identified and three types of mutations for null alleles of *SlPG* were founded at target site SP1 [1 bp insertion (*slpg*-T_1_#2), 4 bp deletion (*slpg*-T_1_#3) and 10 bp deletion (*slpg*-T_1_#5)] (Figure 1E). DNA sequencing peaks showing successful gene editing at the target site are shown in Figure 1F. All types of frameshift mutations induced by CRISPR/Cas9 at the target site generated premature translation termination codons (PTCs) (Supplementary Figure 1). These results demonstrated that CRISRP/Cas9 generates the targeted mutations and loss of function in the tomato *SlPG* gene.

### Potential Off-Target Analysis

According to the predicted result, the one most likely to be the off-target site of SP1 target sites of *SlPG* was found in an exon of Solyc04g015530.2.1 (Figure 1G). They examined potential off-target site only possessed mismatches of 1 bp compared with the on-target guide sequences (Figure 1G). Sequencing analysis demonstrated that sequences of the off-target site in all three homozygous *slpg* lines completely matched the WT sequence in tomato. This result indicates that no mutations were induced at the off-target site.

### Stable Inheritance of Induced Mutations of the Mutants and Morphological Characteristics

To determine whether homozygous *slpg* can transmit the induced mutations and phenotypes to their progenies, a total of 20 T_2_-generation plants of each mutation type deriving from three individuals of corresponding T_1_ *slpg* lines were selected randomly, respectively. The result demonstrated that the targeted mutagenesis of *SlPG* was stably inherited and had maintained consistent mutation types from the T_1_ generation. To assess the consequences of the site-directed mutagenesis in the targeted loci, we examined the morphological characteristics of the plant body and fruits in all three *slpg* mutants and WT plants and detected no morphological differences (Supplementary Figure 2). In addition, some of the agronomic characteristics of all *slpg* mutants and WT plants were also evaluated (Supplementary Table 2). Results indicate that the growth and development of all *slpg* mutants and WT plants are consistent and are not affected by gene editing.

### Expression Patterns of *SlPG* in WT Plants and *slpg*-T_2_ Homozygous Mutants

**FIGURE 2:**
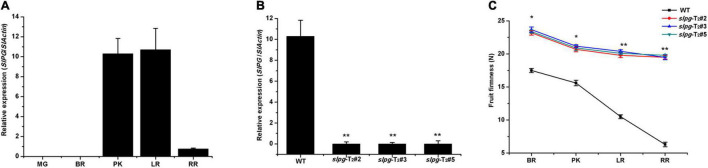
Expression patterns of *SlPG* and phenotype identification of tomato *slpg* mutant. **(A)** Expression of *SlPG* in fruit at different developmental stages: MG, BR, PK, LR, and RR; **(B)** Expression of *SlPG* in WT plants and T_2_ homozygous *slpg* mutants (*slpg*-T_2_#2, *slpg*-T_2_#3, and *slpg*-T_2_#5). Relative transcript levels of *SlPG* were analyzed by qRT-PCR and normalized to *SlActin*. Quantitative PCR data represent means values for three independent biological replicates (*n* = 3); **(C)** Phenotype of enhanced fruit firmness at different fruit development stages in *slpg* mutants. The fruit firmness values represent the means ± standard error (SE) of 20 individual fruit per line at each stage. * and ** indicate significant differences between WT plants and T_2_ homozygous *slpg* mutants (*slpg*-T_2_#2, *slpg*-T_2_#3, and *slpg*-T_2_#5) with *p* < 0.05 and *p* < 0.01, respectively, as determined by *t*-test.

To assess the potential roles of *SlPG* throughout the development of the tomato fruit, we conducted detailed quantitative real-time PCR (qRT-PCR) to examine its transcription in five different fruit development sections [e.g., mature green (MG), BR, PK, LR, and RR]. In WT plants, the expression of *SlPG* was relatively low and negligible in MG fruit. However, the expression level was drastically enhanced from the PK section and reached a maximum in the LR section (Figure 2A). The result showed that the *SlPG* expression level was relatively high in the PK and LR sections while relatively low in the RR section, indicating that *SlPG* functioned mainly in the fruit ripening section and secondary in the fruit softening. In *slpg* mutants, real-time PCR results showed that *SlPG* transcripts were significantly decreased in the PK section when compared with WT plants (Figure 2B).

### Targeted Mutagenesis of *SlPG* Enhances Fruit Firmness and Exhibits Delayed Fruit Softening

**FIGURE 3:**
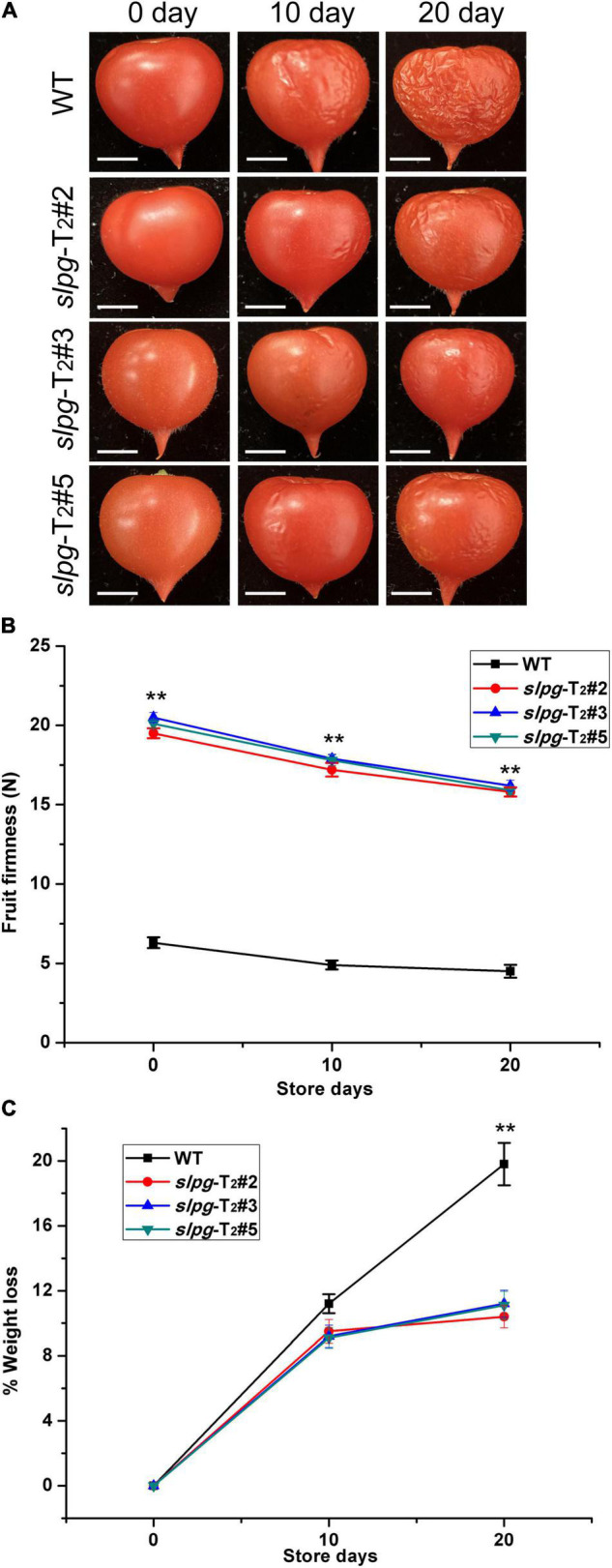
Three *slpg* mutants (*slpg*-T_2_#2, *slpg*-T_2_#3, and *slpg*-T_2_#5) show extended softening time. **(A)** RR fruit from three *slpg* mutants and WT plants after storage at room temperature for 0, 10, and 20 days. Scale bar, 0.5 cm. **(B)** Change of fruit firmness in three *slpg* mutants and WT plants. **(C)** Physiological loss of water (PLW) in three *slpg* mutants and WT plants. The fruit firmness per fruit and PLW were measured after storage of 0, 10, and 20 days. Average and SE (standard error) values were shown for each data point. ** Indicate significant differences between WT plants and three *slpg* mutants with *p* < 0.01, as determined by the *t*-test.

The suitable values for the weight and size of fresh tomato fruit were 4.5 ± 0.2 g and 1.2 ± 0.1 cm, respectively. We next measured the fruit firmness at four different ripening stages (e.g., BR, PK, LR, and RR). The firmness in the WT fruit decreased gradually with fruit ripening, with values of 17.5 ± 0.31, 15.6 ± 0.42, 10.5 ± 0.29, and 6.3 ± 0.34 newton (N) for each stage. In line *slpg*-T_2_#2, the values of fruit firmness for each stage were 23.2 ± 0.34, 20.7 ± 0.38, 19.8 ± 0.29, and 19.5 ± 0.27 N. In line *slpg*-T_2_#3, the values of fruit firmness for each stage were 23.7 ± 0.39, 21.2 ± 0.21, 20.4 ± 0.23, and 19.5 ± 0.38 N. In line *slpg*-T_2_#5, the values of fruit firmness for each stage were 23.4 ± 0.22, 20.9 ± 0.38, 20.1 ± 0.17, and 19.8 ± 0.14 N. All *slpg* mutant lines showed similar symptoms of firmness, implying that the targeted mutagenesis of *SlPG*-enhanced fruit firmness (Figure 2C). The WT and *slpg* mutant fruits were harvested at the RR stage and stored at the greenhouse. WT fruits were wrinkled after 10 days of storage, whereas *slpg* mutant fruits showed similar symptoms of senescence after 20 days of storage. Comparative analysis of images of fruit after storage of 0, 10, and 20 days showed fewer wrinkles for *slpg* mutant than WT fruit (Figure 3A). During storage, fruit firmness was much higher in *slpg* mutant than in WT fruits (Figure 3B). In addition, the *slpg* mutant fruits exhibited lower physiological loss of water than WT fruits (Figure 3C). We compared the fruit softening time between all *slpg* mutants with WT plants and found that *slpg* mutants delayed fruit softening.

### Generation of Transgene-Free Mutant Tomato Lines

**FIGURE 4:**
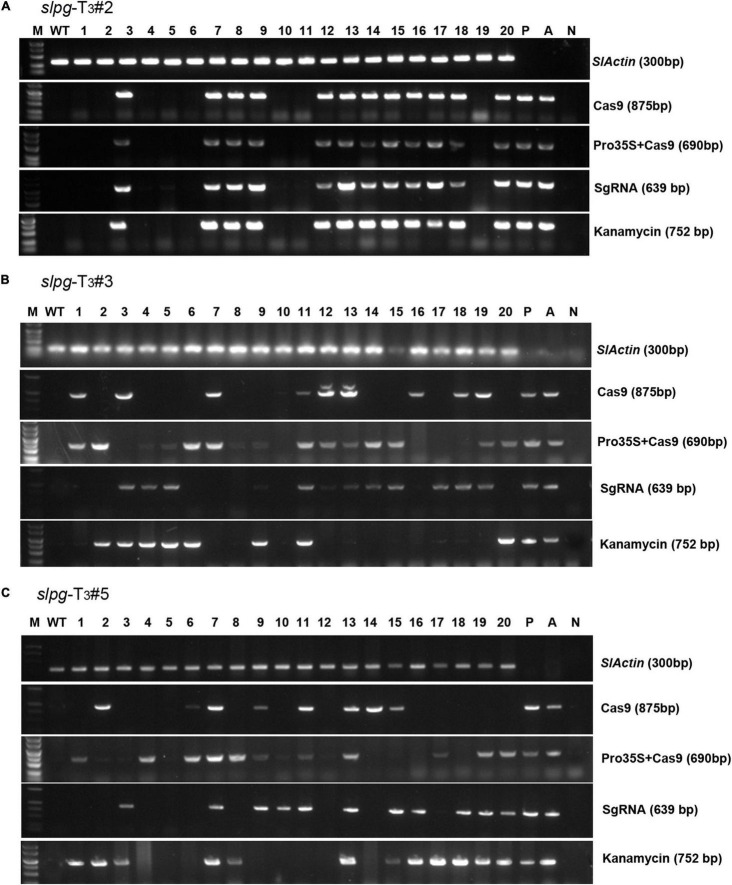
Identifying transgene-free homozygous *slpg* mutants [**(A)** *slpg*-T_3_#2, **(B)** *slpg*-T_3_#3, and **(C)** *slpg*-T_3_#5]. Gel image of PCR products obtained with primer sets for four T-DNA regions of sgRNA/Cas9 vector. *SlActin* (300 bp), part of the tomato Actin coding sequence, was used as a normalization control; Cas9 (875 bp), part of the Cas9 coding sequence; Pro35S + Cas9 (690 bp), region from the downstream of the 35S promoter to the upstream of the Cas9 coding sequence; SgRNA (639 bp), region from the AtU6 promoter to the downstream vector sequence spanning the sgRNA; Kanamycin (752 bp), part of the *nptII* coding sequence; M, DL5000 ladder DNA marker; WT, DNA of WT plant as a template; Lane 1-20, individual mutant lines; P, positive control (sgRNA/Cas9 vector plasmid as template); A, positive control (*Agrobacterium* containing sgRNA/Cas9 vector as template); N, negative control (water as template).

To obtain tomato homozygous lines for endogenous *SlPG* mutations without any transgenic element, “transgene-clean” plants were sought *via* a PCR strategy that used four sets of primer pairs spanning four distinct regions in the T-DNA of sgRNA/Cas9 vectors and one region in the vector backbone. In this assay, we found that 8, 2, and 2 out of 20 T_3_ *slpg* mutants from *slpg*-T_2_#2 (Figure 4A), *slpg*-T_2_#3 (Figure 4B), and *slpg*-T_2_#5 (Figure 4C) and their offspring plants were transgene-clean homozygous *slpg* mutants, respectively.

## Discussion

The CRISPR/Cas9 system has emerged as a robust technology for efficient genome editing and has been successfully applied to many crops (Shan et al., 2013; Upadhyay et al., 2013; Michno et al., 2015; Svitashev et al., 2015). However, some genome editing events do not generate desired specific mutation because of unknown reasons. To overcome this problem, *A. rhizogenes*-mediated transient transformation is an attractive system for assessing the efficiency of sgRNAs in generating InDels at target sites. Different from *A. tumefaciens*, *A. rhizogenes* promote the rapid proliferation of adventitious roots that emerge at the wounding site, which are called hairy roots. The gene-editing efficacy of the sgRNAs in generating mutation at target sites can be evaluated in 15 days by sequencing PCR products amplified from the genomic DNA of pooled hairy roots. Before the whole plant’s stable transform, this transient system can be time-saving, less labor-intensive, and more effective, and many researchers have already applied this system in their works (Pavli and Skaracis, 2010; Li et al., 2019). In this work, we designed five sgRNAs and evaluated the gene-editing efficacy of each sgRNA in the tomato *SlPG* gene. Our data demonstrated that the editing efficacy of sgRNA SP1 is relatively high (75%, in Table 1), and SP1 was chosen and applied in the next stable transformation.

Tomato genomes are *ca*. 900 Mb arranged in 12 chromosomes with 35,768 loci containing protein-coding transcripts^3^. It offers many possible binding sites for CRISPR/Cas9 to generate some additional unwanted mutations. Therefore, a certain risk of off-target activity always remains in the application of CRISPR/Cas9. Previously, several measures, such as choice of suitable nuclease (Fauser et al., 2014), target site choice with CRISPOR (Concordet and Haeussler, 2018), and nuclease delivery method (Hendel et al., 2015), should be considered to minimize the chances for an off-target cleavage to occur. A suitably engineered nucleases can increase the efficiency of targeted mutagenesis. Different types of codon-optimized Cas9 nucleases, including maize codon-optimized (Svitashev et al., 2015), were expressed in our transient assay and the results showed that maize codon-optimized nucleases possess the highest rate of targeted mutagenesis in monocot and dicot plants (paper unpublished). In this study, to avoid potential off-target mutations, a zCas9 nuclease (maize codon-optimized deactivated Cas9), a high specificity score (94) target site SP1 from CRISPOR, and Agrobacterium-mediated transformation were applied.

CRISPR/Cas9-induced mutations were determined from the T_0_ to T_1_ generation. All T_0_ heterozygous biallelic and homozygous *slpg* edited lines could transmit the targeted mutations to the T_1_ generation. We still obtained some novel targeted mutagenesis in T_1_ generation from T_0_ heterozygous monoallelic *slpg* lines. In these lines, CRISPR/Cas9-induced mutation exists only in one allele and the other allele without any mutation, and the targeted mutations of the *SlPG* allele induced by CRISPR/Cas9 were stably inherited and maintained consistent mutation types from T_0_ to T_1_ generation. At the same time, the other allele without any mutations can be induced by CRISPR/Cas9 and generate novel targeted mutagenesis in the T_1_ generation. These results demonstrated that CRISPR/Cas9 can induce targeted mutagenesis in the next generation if monoallelic edits are in the previous generation. This phenomenon is consistent with the previous research on soybean (Bai et al., 2020).

Fruit softening is one of the most characteristic physiological processes that occur during the ripening of fleshy fruits (Garcia-Gago et al., 2009). A softening process results from the degradation of cell wall polymers by the action of the cell wall-associated hydrolytic enzymes. In the past few decades, many enzymes, such as pectate lyase (PL) (Uluisik et al., 2016; Yang et al., 2017), β-galactosidase (Smith et al., 2002), expansin (Brummell et al., 1999), and GA2-oxidase (Li et al., 2020), have been studied as candidates for delaying fruit softening, and some changes were observed in fruit softening. For example, the PL gene has already been chosen to manipulate fruit softening in tomatoes (Marín-Rodríguez et al., 2002; Yang et al., 2017), bananas (Payasi and Sanwal, 2003), and mangos (Chourasia et al., 2006). Among the cell hydrolytic enzymes involved in fruit softening, PG is the best and the earliest characterized (Brady et al., 1982). In 1986, the *PG* gene was first cloned and sequenced (Grierson et al., 1986). In 1994, The FLAVR SAVR™ tomato is the first genetically engineered commodity to be sold in commerce (Kramer and Redenbaugh, 1994). By 2000, the members of the homologs of *SlPG* have reached 7 (Hong and Tucker, 2000). The last cloned *PG* gene, *TPG7*, is highly expressed in pistils and shares 39% sequence identity with the tomato fruit *PG* genes. The *PG* gene locus, solyc10g080210, was proved to be highly expressed in tomato fruit, especially in fruit PK and LR stage (Figure 2A). These results were also basically consistent with the data obtained from the TomExpress platform^4^, thereby further implying a possible crucial role of *SlPG* during fruit ripening and softening. The FLAVR SAVR™ tomato was developed by using antisense RNA to suppress the expression of PG in ripening tomato fruit. Subsequently, this antisense RNA strategy was applied to other fruits to produce firmer fruits (Garcia-Gago et al., 2009; Zhang S. et al., 2019; Huang et al., 2020). However, these commodities were developed through a transgenic approach, which may generate negative results, such as the disruption of plant endogenous genes or exogenous gene silencing (Napoli et al., 1990). Although no scientific evidence suggests that genetically modified organism (GMO) products are harmful to human health, debates about GMO safety continue. Previously, many transgene-free targeted mutagenesis plants, such as soybean (Cai et al., 2018), rice (Li et al., 2016), wheat (Zhang et al., 2016), and tomato (Soyk et al., 2017), have been generated by CRISPR/Cas9. In this study, CRISPR/Cas9 was applied to induce targeted *SlPG* mutagenesis, which could delay fruit softening without any exogenous gene fragment. Moreover, CRISPR/Cas9 was proven to enhance fruit firmness without compromising other fruit qualities (Li et al., 2020), indicating that the targeted mutagenesis of the *SlPG* gene in tomatoes could delay fruit softening without impacting the tomato flavor, aroma, taste, and nutritional value. This conclusion was supported by Kramer and Redenbaugh’s research (Kramer and Redenbaugh, 1994). This is a key target characteristic in modern tomato breeding.

## Data Availability Statement

The datasets presented in this study can be found in online repositories. The names of the repository/repositories and accession number(s) can be found in the article/Supplementary Material.

## Author Contributions

HN and GX designed the project. HN and YS performed the experiment. HN and XG analyzed the data. HN wrote the manuscript. GX made suggestions for improving the procedure and proofread the manuscript. All authors read and approved the final manuscript.

## Conflict of Interest

The authors declare that the research was conducted in the absence of any commercial or financial relationships that could be construed as a potential conflict of interest.

## Publisher’s Note

All claims expressed in this article are solely those of the authors and do not necessarily represent those of their affiliated organizations, or those of the publisher, the editors and the reviewers. Any product that may be evaluated in this article, or claim that may be made by its manufacturer, is not guaranteed or endorsed by the publisher.

## Footnotes

1
www.phytozome.net/

2
http://crispor.tefor.net/

3
https://phytozome-next.jgi.doe.gov/info/Slycopersicum_ITAG3_2

4
http://tomexpress.toulouse.inra.fr/
