# Supplementary material for: CRISRP/Cas9-Mediated Targeted Mutagenesis of Tomato Polygalacturonase Gene (SlPG) Delays Fruit Softening
Source: Front Plant Sci. 2022 May 19;13:729128. doi: 10.3389/fpls.2022.729128 (PMC9162796; doi:10.3389/fpls.2022.729128)
Supplement: Supplementary file 1 [file Data_Sheet_1.docx]

Supplementary Material

CRISRP/Cas9-mediated targeted mutagenesis of tomato polygalacturonase gene (*SlPG*) delays fruit softening

Hongmei Nie^1^, Yu Shi^1^, Xueqing Geng^2^, Guoming Xing^1*^

^1^College of Horticulture / Collaborative Innovation Center of Improving Quality and Increasing Profits for Protected Vegetables in Shanxi, Shanxi Agricultural University, Taigu, 030801, Shanxi Province, China.

^2^School of Agriculture and Biology, Shanghai Jiao Tong University, Shanghai, 200240, China.

*** Correspondence:**Guoming Xing
kaiander@163.com

**Supplemental Table 1** Primer sequences used in the present study

| Primer name | Primer sequence | Size (bp) | Purpose |
| --- | --- | --- | --- |
| SP1-F | 5’-TAACCATATTGACCAACTCAAACC-3’ | 575 | To detect mutagenesis induced by sgRNA SP1 |
| SP1-R | 5’-TGACTTTCCTCGCTTACGAG-3’ |  |  |
| SP2-F | 5’-TAACCATATTGACCAACTCAAACC-3’ | 575 | To detect mutagenesis induced by sgRNA SP2 |
| SP2-R | 5’-TGACTTTCCTCGCTTACGAG-3’ |  |  |
| SP3-F | 5’-CCTCGACAAAACGACAATGC-3’ | 545 | To detect mutagenesis induced by sgRNA SP3 |
| SP3-R | 5’-GCTAATTCATAGCTAAGTTGCTC-3’ |  |  |
| SP4-F | 5’-CAAAGTAAACGAAATAACAATAGG-3’ | 628 | To detect mutagenesis induced by sgRNA SP4 |
| SP4-R | 5’-AAGGGGTAATAGGTCCTGCC-3’ |  |  |
| SP5-F | 5’-CAAAGTAAACGAAATAACAATAGG-3’ | 628 | To detect mutagenesis induced by sgRNA SP5 |
| SP5-R | 5’-AAGGGGTAATAGGTCCTGCC-3’ |  |  |
| OFF-F | 5’-ATGGAGAAATTCAATGAAGAAG-3’ | 423 | To examine potential off-target site in exon of Solyc04g015530 |
| OFF-R | 5’-ACATTTAAGCCTTTACAACC-3’ |  |  |
| *SlActin*-F | 5’-TGTCCCTATCTATGAAGGTT-3’ | 300 | Housekeeping gene used as internal control to qRT-PCR analysis |
| *SlActin*-R | 5’-AAGAGTACCTCTGGGCAACG-3’ |  |  |
| *SlPG*-F | 5’-TGCATTTGAGCAAGCATGGA-3’ | 309 | To analysis the gene expression level by qRT-PCR |
| *SlPG*-R | 5’-TCCCTGCATGGCAGTGATTT-3’ |  |  |
| Cas9-F | 5’-GTTCATCAAGCCGATTCTGG-3’ | 875 | To amplify part of Cas9 coding sequence |
| Cas9-R | 5’-GCTTCCTGCTCAGCCTCCC-3’ |  |  |
| Pro35S+Cas9-F | 5’-GACGTAAGGGATGACGCACA-3’ | 690 | To amplify the region from the 35S promoter to the upstream of the Cas9 coding sequence |
| Pro35S+Cas9-R | 5’-TCGTGCTTCTTATCCTCCTC-3’ |  |  |
| SgRNA-F | 5’-GACGTAAGGGATGACGCACA-3’ | 639 | To amplify region from the AtU6-26 promoter to the downstream vector sequence spanning the sgRNA |
| SgRNA-R | 5’-CTGAAGTCCAGCTGCCAGAA-3’ |  |  |
| Kanamycin-F | 5’-GAAGAACTCGTCAAGAAGGC-3’ | 752 | To amplify part of NPTII coding sequence in vector |
| Kanamycin-R | 5’-CTTGGGTGGAGAGGCTATTCG-3’ |  |  |

**Supplemental Table 2** Evaluation of all three *slpg* mutants and WT plant for agronomic characteristics

| **Parameter** | WT | *slpg*-T_2_#2 | *slpg*-T_2_#3 | *slpg*-T_2_#5 |
| --- | --- | --- | --- | --- |
| Seed germination (%) | 76.3±9.2 | 74.3±5.7 | 70.3±6.4 | 72.1±4.8 |
| 100 seed weight (g) | 0.2±0.0 | 0.2±0.0 | 0.2±0.0 | 0.2±0.0 |
| Days from anthesis to breaker (days) | 41.2±2.1 | 40.4±1.7 | 39.8±2.7 | 40.5±1.8 |
| Plant height (cm) | 25.3±4.2 | 22.3±3.2 | 24.3±2.8 | 23.3±3.7 |
| Fruit weight (g)（at the RR stage） | 4.5±0.2 | 4.4±0.3 | 4.4±0.2 | 4.5±0.1 |
| Fruit size (cm) （at the RR stage） | 1.2±0.1 | 1.2±0.1 | 1.2±0.1 | 1.2±0.1 |
| Fruit yield/plant (g) | 60.4±5.7 | 62.4±7.8 | 58.7±5.7 | 61.3±6.2 |

**Supplemental Figure 1** Alignment of predicted amino acid sequences for the *SlPG* gene in the mutants (*slpg*-T_1_#2, *slpg*-T_1_#3, and *slpg*-T_1_#5) and WT (SlPG).

**
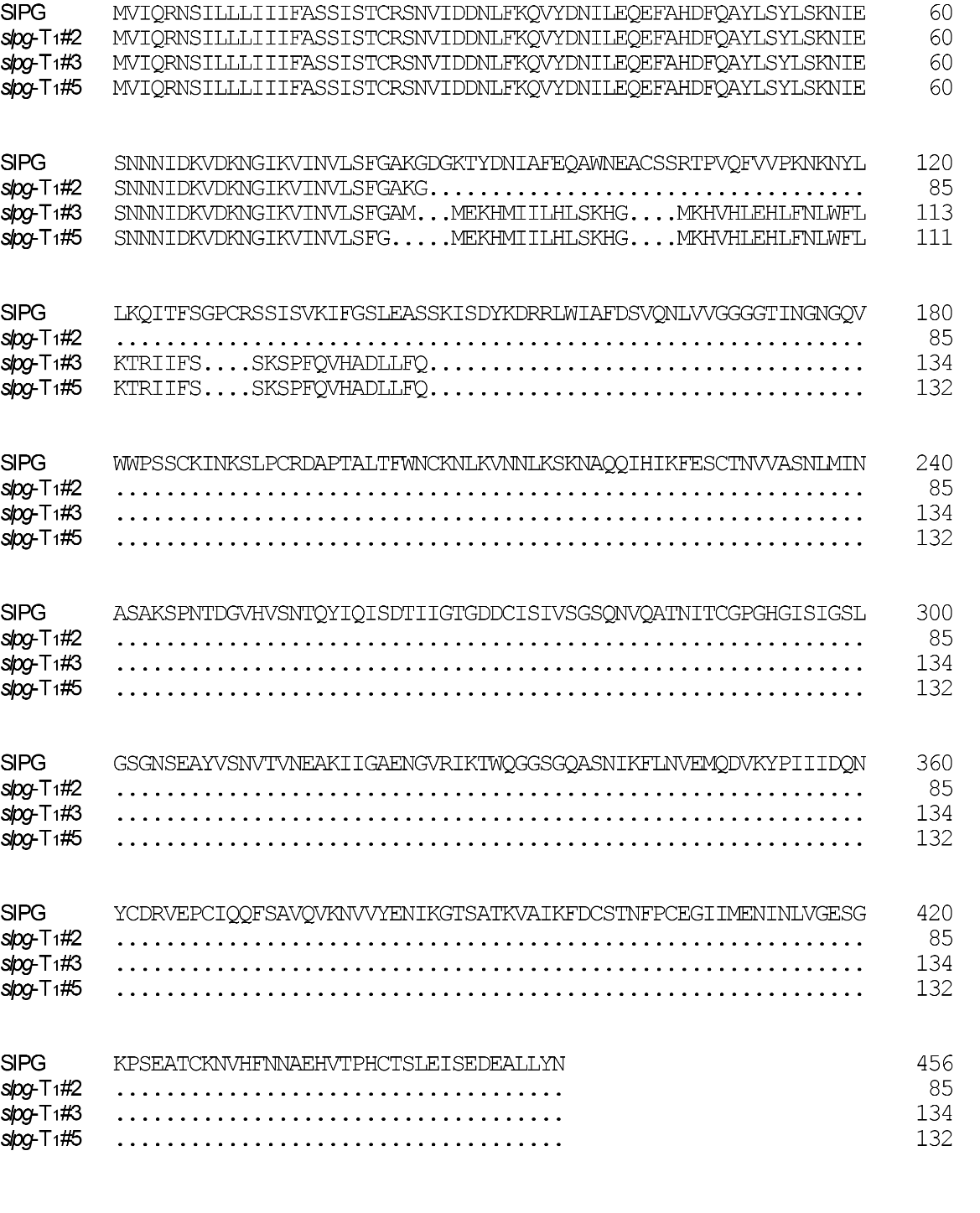
**

**Supplemental Figure 2** Morphological characteristics of plant body of the mutants (*slpg*-T_2_#2, *slpg*-T_2_#3, and *slpg*-T_2_#5) and WT plant. Scale bar, 10 cm.

**
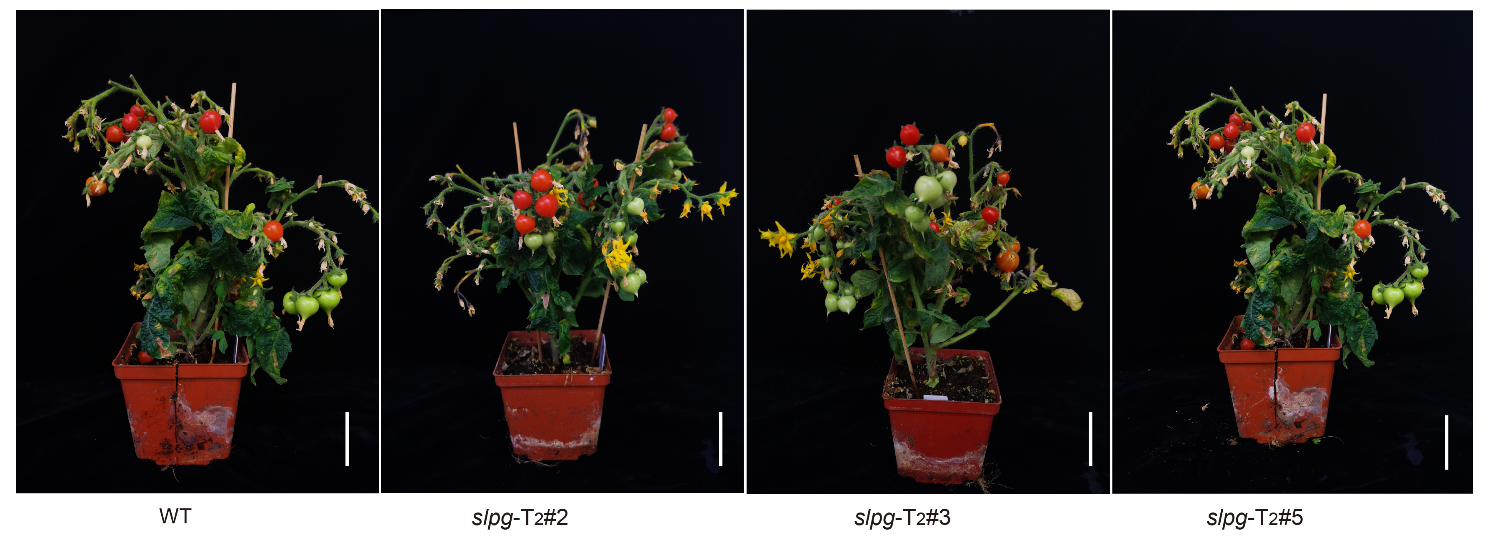
**
